# Gold and Nickel Extended Thiophenic-TTF Bisdithiolene Complexes

**DOI:** 10.3390/molecules23020424

**Published:** 2018-02-14

**Authors:** Rafaela A. L. Silva, Bruno J. C. Vieira, Marta M. Andrade, Isabel C. Santos, Sandra Rabaça, Elsa B. Lopes, Joana T. Coutinho, Laura C. J. Pereira, Manuel Almeida, Dulce Belo

**Affiliations:** C^2^TN, Centro de Ciências e Tecnologias Nucleares, Instituto Superior Técnico, Universidade de Lisboa, E.N. 10 ao km 139.7, 2695-066 Bobadela LRS, Portugal; rafaela@ctn.tecnico.ulisboa.pt (R.A.L.S.); brunovieira@ctn.tecnico.ulisboa.pt (B.J.C.V.); marta.m.s.andrade@gmail.com (M.M.A.); icsantos@ctn.tecnico.ulisboa.pt (I.C.S.); sandrar@ctn.tecnico.ulisboa.pt (S.R.); eblopes@ctn.tecnico.ulisboa.pt (E.B.L.); coutinho.joana@ctn.tecnico.ulisboa.pt (J.T.C.); lpereira@ctn.tecnico.ulisboa.pt (L.C.J.P.); malmeida@ctn.tecnico.ulisboa.pt (M.A.)

**Keywords:** gold bisdithiolene complexes, nickel bisdithiolene complexes, thiophenetetrathiafulavalenedithiolate ligands, single component molecular conductors

## Abstract

Gold and nickel bisdithiolene complexes with methyl and *tert*-butyl substituted thiophenetetrathiafulavalenedithiolate ligands (α-mtdt and α-tbtdt) were prepared and characterized. These complexes were obtained, under anaerobic conditions, as tetrabutylammonium salts. The diamagnetic gold monoanion (*n*-Bu_4_N)[Au(α-mtdt)_2_] (**3**) and nickel dianionic species (*n*-Bu_4_N)_x_[Ni(α-mtdt)_2_] (x = 1,2) (**4**) were similar to the related non-substituted extended thiophenic-TTF (TTF = tetrathiafulvalene) bisdithiolenes. However the introduction of the large, bulky substituent *tert*-butyl, led to the formation of a Au (I) dinuclear complex, (*n*-Bu_4_N)_2_[Au_2_(α-tbtdt)_2_] (**5**). The neutral methyl substituted gold and nickel complexes were easily obtained through air or iodine exposure as polycrystalline or amorphous fine powder. [Au(α-mtdt)_2_] (**6**) and [Ni(α-mtdt)_2_] (**7**) polycrystalline samples display properties of a metallic system with a room temperature electrical conductivity of 0.32 S/cm and ≈4 S/cm and a thermoelectric power of ≈5 µV/K and ≈32 µV/K, respectively. While [Au(α-mtdt)_2_] (**6**) presented a Pauli-like magnetic susceptibility typical of conducting systems, in [Ni(α-mtdt)_2_] (**7**) large magnetic susceptibilities indicative of high spin states were observed. Both electric transport properties and magnetic properties for gold and nickel [M(α-mtdt)_2_] are indicative that these compounds are single component molecular conductors.

## 1. Introduction

**Scheme 1:**
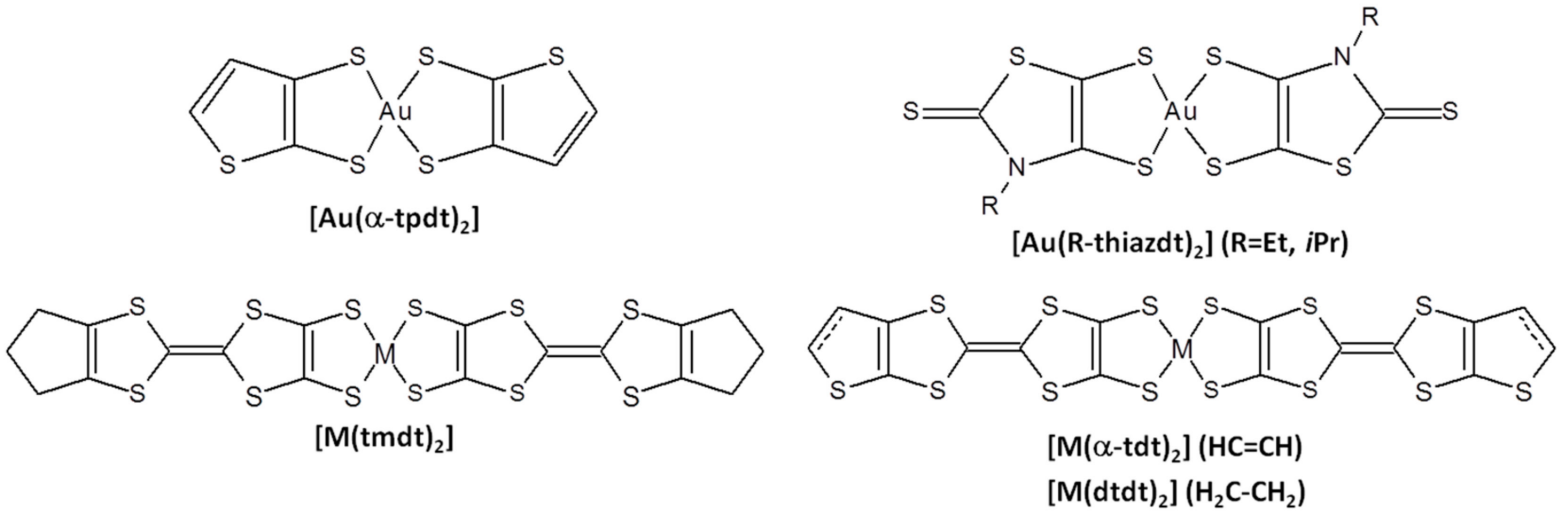
Molecular diagrams of selected single component molecular metals (SCMMs).

High electrical conductivity and properties typical of a metal in a compound based on a neutral molecular species were initially reported by our group in the neutral gold complex [Au(α-tpdt)_2_] (α-tpdt = 2,3-thiophenedithiolate) (Scheme 1) [1] and was soon followed by [Ni(tmdt)_2_] (tmdt = trimethylenetetrathiafulvalenedithiolate) (Scheme 1), an extended TTF-bisdithiolene type of complex, reported by A. Kobayashi and co-workers [2]. Both were milestones in the development of single component molecular metals (SCMM). [Au(α-tpdt)_2_] presented a room temperature electrical conductivity of σ_RT_ = 7 S/cm as a polycrystalline sample while single crystals of [Ni(tmdt)_2_] presented σ_RT_ = 400 S/cm [1,2,3]. These neutral complexes enabled the establishment of a new paradigm of molecular conductors where free carriers and metallic properties can be generated in molecular compounds based on a neutral single-component by a mechanism characteristic of semi-metals. The requirements for such properties were soon established as a small HOMO-LUMO energy gap and strong 3D intermolecular interactions [4,5,6].

Following these initial reports several SCMM were subsequently reported. A large number of them are transition metal bisdithiolene complexes with extended π ligands incorporating a TTF framework since bisdithiolene complexes tend to present a small HOMO-LUMO gap [4,7] and extended ligands favor efficient 3D interactions. A limitation of these neutral complexes is their relatively low solubility which severely limits their isolation as single crystals and further characterization [8,9,10]. The incorporation of alkyl-chain substituents in these ligands is expected to enhance the solubility of the neutral complexes. Although the introduction of large, bulky substituents is known to improve charge mobility in materials for organic field effect transistors [11,12], it can substantially limit the solid-state intermolecular interactions and decrease electrical conductivity [13,14]. This was not observed for example when the ethyl group of [Au(Et-thiazdt)_2_] SCMM (Scheme 1) [15] was substituted by the bulkier isopropyl group, [Au(*i*Pr-thiazdt)_2_] (Scheme 1) [16]. In this case the system found a way to favour strong intermolecular overlap interactions, and there was an increase of conductivity from σ_RT_ = 0.33 S/cm in the ethyl substituted to σ_RT_ = 5 S/cm for the isopropyl incorporation [15,16].

Here we report the preparation of a Au (I) dinuclear monoanionic complex, (*n*-Bu_4_N)_2_[Au_2_(α-tbtdt)_2_] and the synthesis and characterization of two neutral gold and nickel complexes, [M(α-mtdt)_2_]^0^ (M = Au, Ni), all based on extended alkyl-substituted thiophenic-TTF bisdithiolate ligands (Scheme 1). Besides enlarging the SCMM family of compounds, the characterization of these salts allowed the study of steric effects on electric conductivity when compared with the transport properties of related non-substituted complexes, [M(α-tdt)_2_]^0^ and [M(dtdt)_2_]^0^ (α-tdt = thiophenetetrathiafulvalenedithiolate, dtdt = dihydrothiophenetetrathiafulvalenedithiolate; M = Au and Ni) [17]. SCMM are promising candidates as components in electronic devices, being able to be processed using solution techniques they can be used, for example as conductive inks [18].

## 2. Results and Discussion

### 2.1. Synthesis

**Scheme 2:**
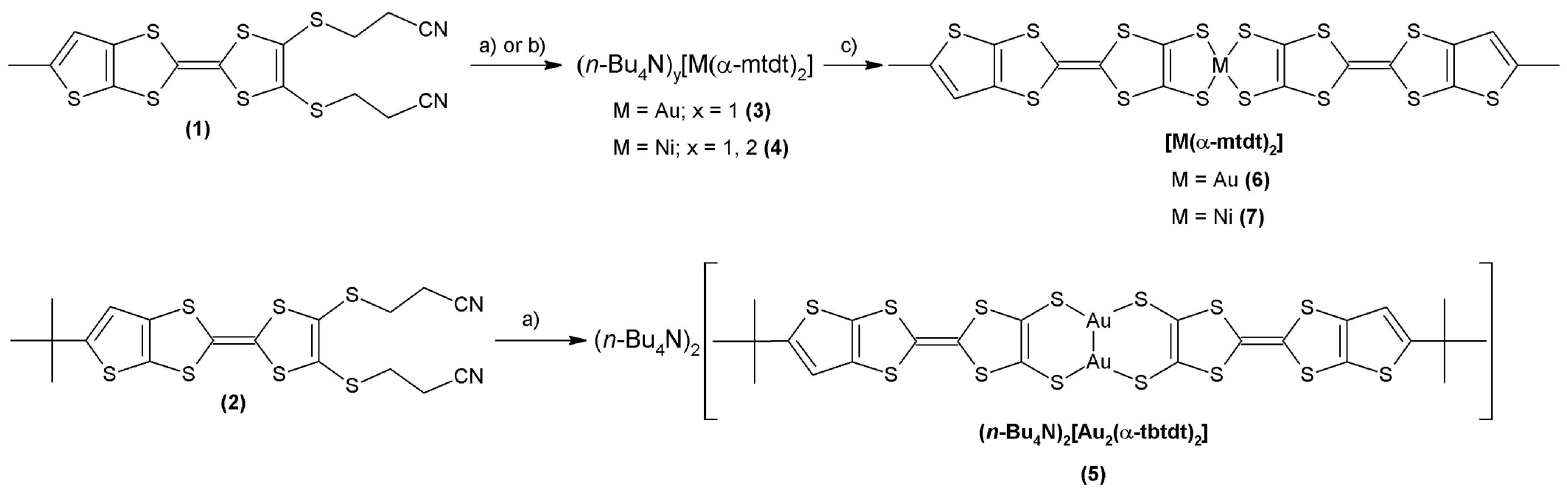
Preparation of extended transition metal bisdithiolenes **3**–**7**: (**a**) in MeONa; KAuCl_4_ in MeOH; *n*-Bu_4_NBr in MeOH; (**b**) in MeONa; NiCl_2_.6H_2_O in MeOH; *n*-Bu_4_NBr in MeOH; (**c**) by air exposure or treatment with an iodine solution.

The synthesis of the gold and nickel complexes **3**–**7** were performed following a general common procedure frequently used for the preparation of similar bisdithiolene complexes [19] (Scheme 2). The TTF donors non-symmetrically fused with substituted thiophene moeieties **1** and **2**, that have been recently reported by us [20], are employed here as potential ligand precursors for the formation of extended transition metal bisdithiolenes. The thiophenic-TTF bisdithiolate ligands α-mtdt and α-tbtdt were obtained in solution from the corresponding precursors, **1** and **2**, from a hydrolytic cleavage with sodium methoxide in methanol followed by the immediate addition of tetrachloroaurate or nickel chloride, leading to the precipitation of the corresponding tetrabutylammonium salts after treatment with *n*-Bu_4_NBr. In the particular case of the nickel compound **4**, in spite of strict anaerobic conditions, elemental analysis indicated a variable cation/anion stoichiometry (*n*-Bu_4_N)_x_[Ni(α-mtdt)_2_] (x = 1–2), similarly to what was observed in the analogous salts (*n*-Bu_4_N)_x_[Ni(dtdt)_2_] and (*n*-Bu_4_N)_x_[Ni(α-tdt)_2_] [17]. The tetrabutylammonium salt of the monoanionic gold complex **5** presented a higher solubility than related unsubstituted compounds, however the attempt to prepare the transition metal complex lead instead to a Au(I) complex, (*n*-Bu_4_N)_2_[Au_2_(α-tbtdt)_2_]. While not common, the formation of this type of Au (I) dinuclear complex has been found with other bisdithiolene ligands under specific experimental conditions [21].

Oxidation of the anionic Au and Ni complexes to their neutral states **6** and **7**, were carried out by treatment of the anionic complexes **3** and **4** with an iodine solution, or simple by air exposure in the case of the nickel complex **7**. All neutral complexes were obtained as dark fine powders with poor solubility in common solvents, which so far did not allow the growth of single crystal suitable for X-ray diffraction. Small crystals suitable for X-ray diffraction studies could be isolated from the preparations of gold tetrabutylammonium salts **3** and **5,** which allowed the determination of their structure. In the case of the nickel tetrabutylammonium salt **4,** the crystals were too small precluding X-ray structural analysis.

### 2.2. Electrochemical Studies

Cyclic voltammetry studies were limited by low stability in solution of the compounds. Even so it was possible to conclude that there are several processes resulting from species in chemical equilibria (Figures S1 and S2). As already referred above, in spite of the strict anaerobic conditions in the synthesis of the nickel complex, the samples were a mixture of two anionic species, which is similar to what was observed in the analogous salts (*n*-Bu_4_N)_x_[Ni(dtdt)_2_] and (*n*-Bu_4_N)[Ni(α-tdt)_2_] and is probably due to the low and close oxidation potentials of the monoanionic and neutral states (Figure S1). This is in agreement with the fact that when exposed to air the nickel salt quickly oxidizes to the neutral state under the form of dark powder. The gold complex **3** (Figure S1) presents a similar behavior as the previous reported (*n*-Bu_4_N)[Au(-α-tdt)_2_] and (*n*-Bu_4_N)[Au(dtdt)_2_] [17]. Likewise, the oxidation of [Au(α-mtdt)_2_]^−^ to a neutral state only occurs in the presence of a strong oxidant like iodine, indicating a higher oxidation potential of the monoanionic gold complex to the neutral species. Despite being instable in solution the preliminary voltammetry studies of the nickel compound **4** showed two close redox processes below −500 mV. In the case of the gold complex it presents a process centered at ≈616 mV (conversion to the neutral state) and another one at negative potentials (≈−500 mV). In (*n*-Bu_4_N)_2_[Au_2_(α-tbtdt)_2_] (**5**), several dependent oxidation processes were observed above −250 mV, which can be indicative of a complex redox process mechanism in solution of this bimetallic specie (Figure S2).

### 2.3. X-ray Structural Analysis

Small size crystals could be isolated from the gold tetrabutylammonium salts **3** and **5** which allowed the determination of their X-ray structure (crystal structure of ligand precursor **1** in Supplementary Materials). In the case of the nickel tetrabutylammonium salt **4,** the crystals were too small and the X-ray diffraction peaks were very weak, which did not allowed the single crystal X-ray structure analysis. Nevertheless the X-ray powder pattern of **4** when compared to the X-ray powder patter simulation of **3** could evidence similar packing pattern (Figure S3).

**Figure 1:**
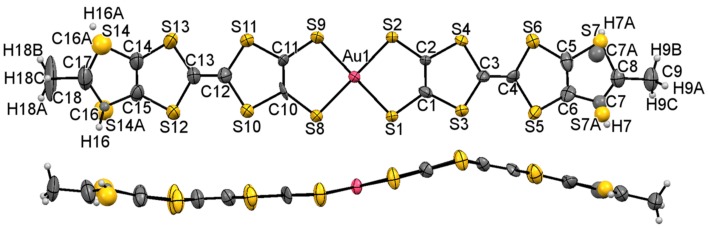
ORTEP and atomic numbering schemes (top and side views) of [Au(α-mtdt)_2_] monoanion in the crystal structure of (*n*-Bu_4_N)[Au(α-mtdt)_2_] (**3**), with thermal ellipsoids drawn at 50% probability level.

The gold compound **3** was found to crystallize in the triclinic system, space group *P*-1. The asymmetric unit cell contains one (*n*-Bu_4_N)^+^ cation and one [Au(α-mtdt)_2_]^−^ anion, both at general positions (Table S1). The cation presents a typical geometry while [Au(α-mtdt)_2_]^−^ shows considerable deviations from planarity, with exception of the flat square planar coordination geometry at the AuS_4_ core, which has Au–S bond lengths typical of monoanionic Au bisdithiolene complexes (*d*_Au-S_ = 2.317 Å). The two ligands present considerably different distortions, one with a boat type and the other a large bending at S4 and S3 sulfur atoms, with a 26° dihedral angle between the TTF moiety and the AuS_4_ core average plane (Figure 1). One of the four butyl chains in the tetrabutylammonium cation presents a disorder in the C32 atom over two positions with an occupation factor of 76–24% (C32–C32A). [Au(α-mtdt)_2_] monoanion also displays the usual *cis-trans* disorder as denoted by the thiophenic sulfur atoms S7 and S14 in two possible positions with occupation factors of 51–49% (in both S7/C7-S7A/C7A and S14/C16-S14A/C16A).

**Figure 2:**
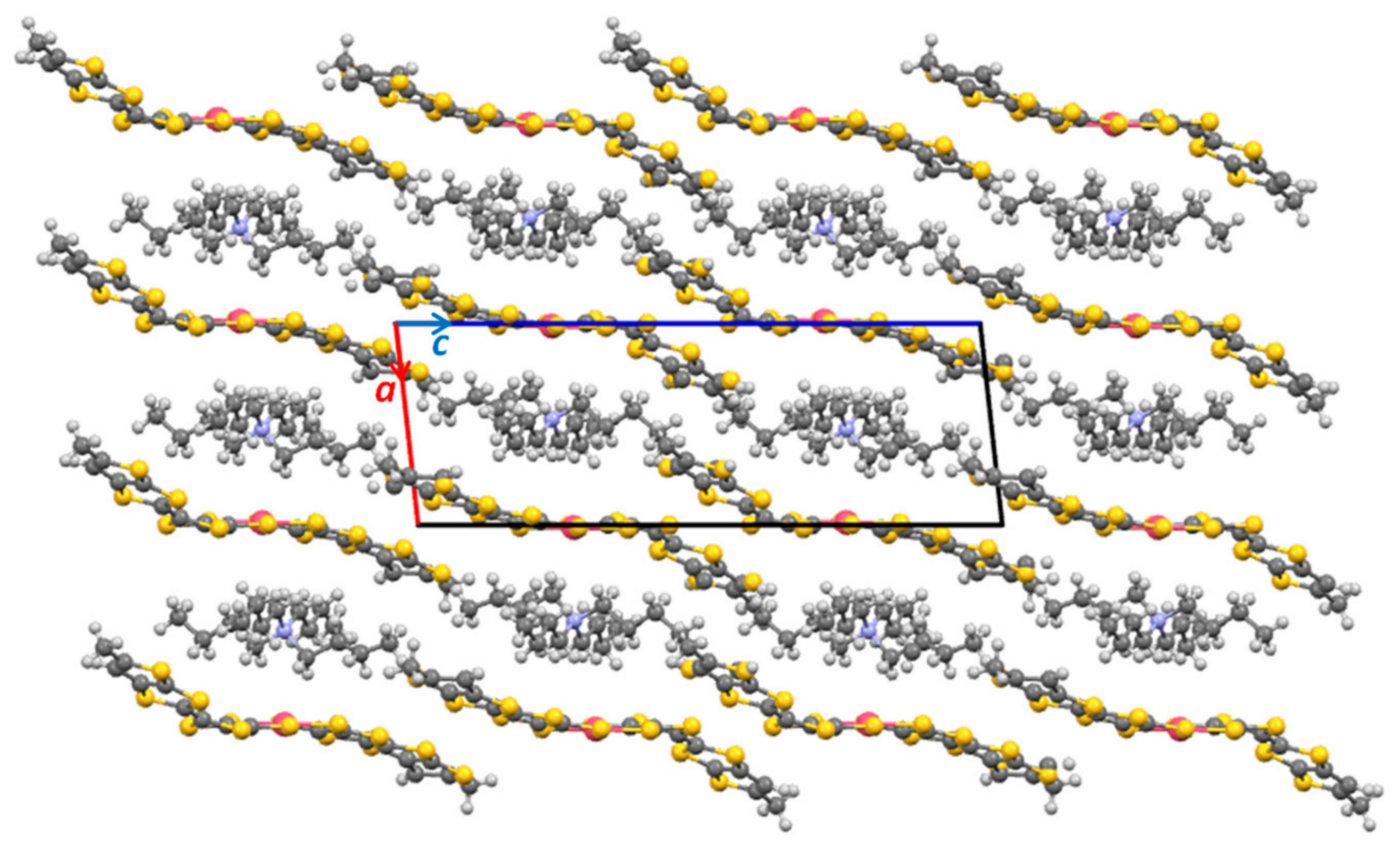
(*n*-Bu_4_N)[Au(α-mtdt)_2_] (**3**) crystal structure viewed along *b* axis.

**Figure 3:**
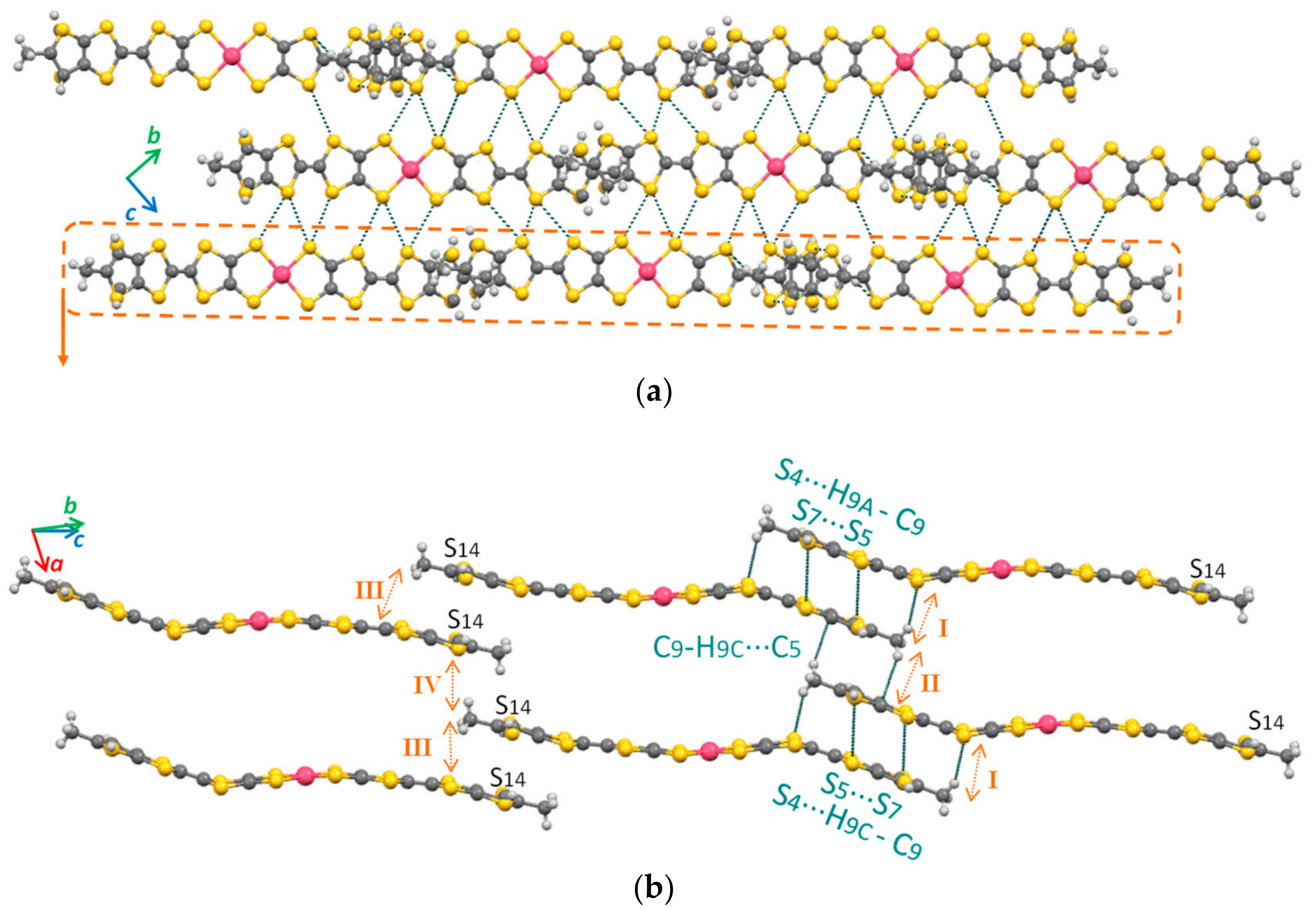
Anions in the crystal structure of (*n*-Bu_4_N)[Au(α-mtdt)_2_] (**3**): (**a**) view perpendicular to one anionic layer with side-by-side contacts; (**b**) view perpendicular to anionic chains with thiophenic ring contacts in a chain and between different layers with mean plane distances of 3.485 Å (**I**), 3.588 Å (**II**), 3.869 Å (**III**) and 3.674 Å (**IV**).

Figure 2 illustrates (*n*-Bu_4_N)[Au(α-mtdt)_2_] (**3**) crystal packing pattern consisting in cationic and monoanionic layers parallel to *b, c* alternating, along the *a* axis. The monoanionic layers are composed of domino-like chains of the complexes, running parallel to *c*. Within the anionic layers the chains are connected, side-by-side, by a network of S···S contacts, as shown in Figure 3a (Table S2). Along the chains the molecules overlap through the peripheral thiophenic rings in two distinct ways: in one side they are connected through short S···S contacts and hydrogen bonds (S5···S7 and S4···H9A-9), with a mean plane distance of 3.485 Å (interaction “I” in Figure 3b); in the other side the molecules have a smaller overlap with no short contacts and a intermolecular average plane distance of 3.869 Å (interactions “III” in Figure 3b). Despite the interleaving of cationic layers, molecules in consecutive monoanionic layers are only 3.588 Å and 3.674 Å apart (shortest distance between molecular mean average plane: interactions “II” and “IV” in Figure 3b). The cationic and monoanionic layers are connected through several S···H–C hydrogen bonds (Table S2).

It is worth referring that the crystal structure of (*n*-Bu_4_N)[Au(α-mtdt)_2_] (**3**) presents a packing pattern similar to those of the previously described related compounds (*n*-Bu_4_N)[Au(dtdt)_2_] and (*n*-Bu_4_N)[M(α-tdt)_2_] (M = Au, Ni), all with a strong segregation of anions and cations in alternating layers and with the anions arranged in domino-like chains [10,17].

**Figure 4:**
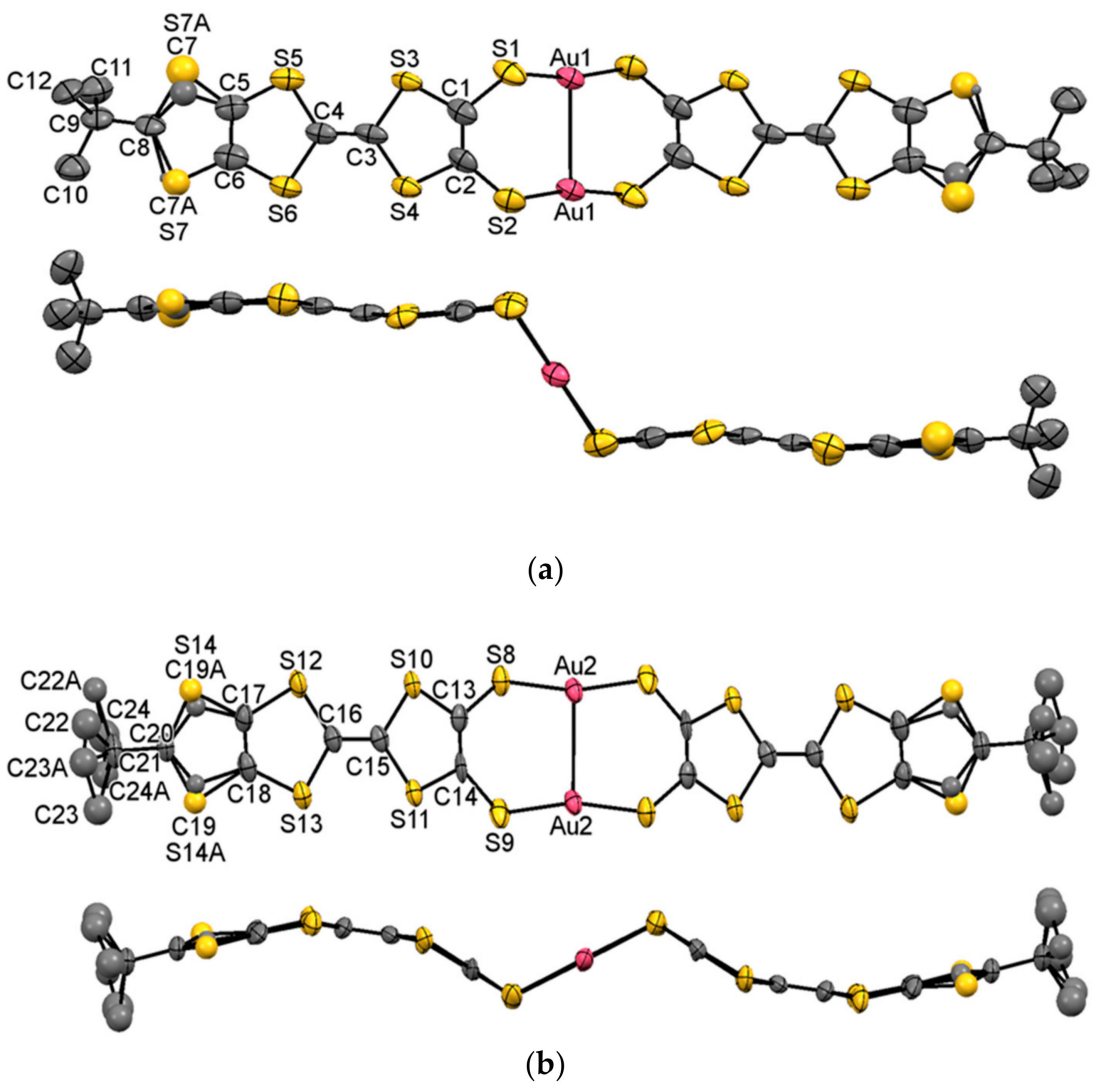
ORTEP and atomic numbering schemes (top and side views) of [Au(α-tbtdt)_2_] anions Au1 (**a**) and Au2 (**b**) in the crystal structure of (*n*-Bu_4_N)_2_[Au_2_(α-tbtdt)_2_] (**5**), with thermal ellipsoids drawn at 50% probability level. Hydrogen atoms were omitted for clarity.

Compound **5**, (*n*-Bu_4_N^+^)_2_[Au_2_(α-tbtdt)_2_]^2−^, crystallizes in the triclinic system, space group *P*-1. The asymmetric unit cell contains two (*n*-Bu_4_N)^+^ cations at general positions and two [Au_2_(α-tbtdt)_2_] complexes, both at an inversion center (Tables S3 and S4). The (*n*-Bu_4_N)^+^ cations present a typical geometry with disorder in one of the four butyl chains, where the C35 and C36 atoms were refined in two positions with site occupancy of 54–46%. Both gold complexes, present an almost perfect planar geometry of the Au_2_S_4_ core, folded relatively to the remaining ligand. The ligands in Au1 complex are essentially planar, while in Au2 they are significantly bended with a boat type distortion (Figure 4) and Au2 also presents some disorder in the *tert-*butyl group. Both complexes display a disorder in the thiophenic ring with the sulfur atom over two possible positions with occupation factors of 74–26% (S7/C7-S7A/C7A) for Au1 and of 44–56% (S14/C19-S14A/C19A) for Au2.

**Figure 5:**
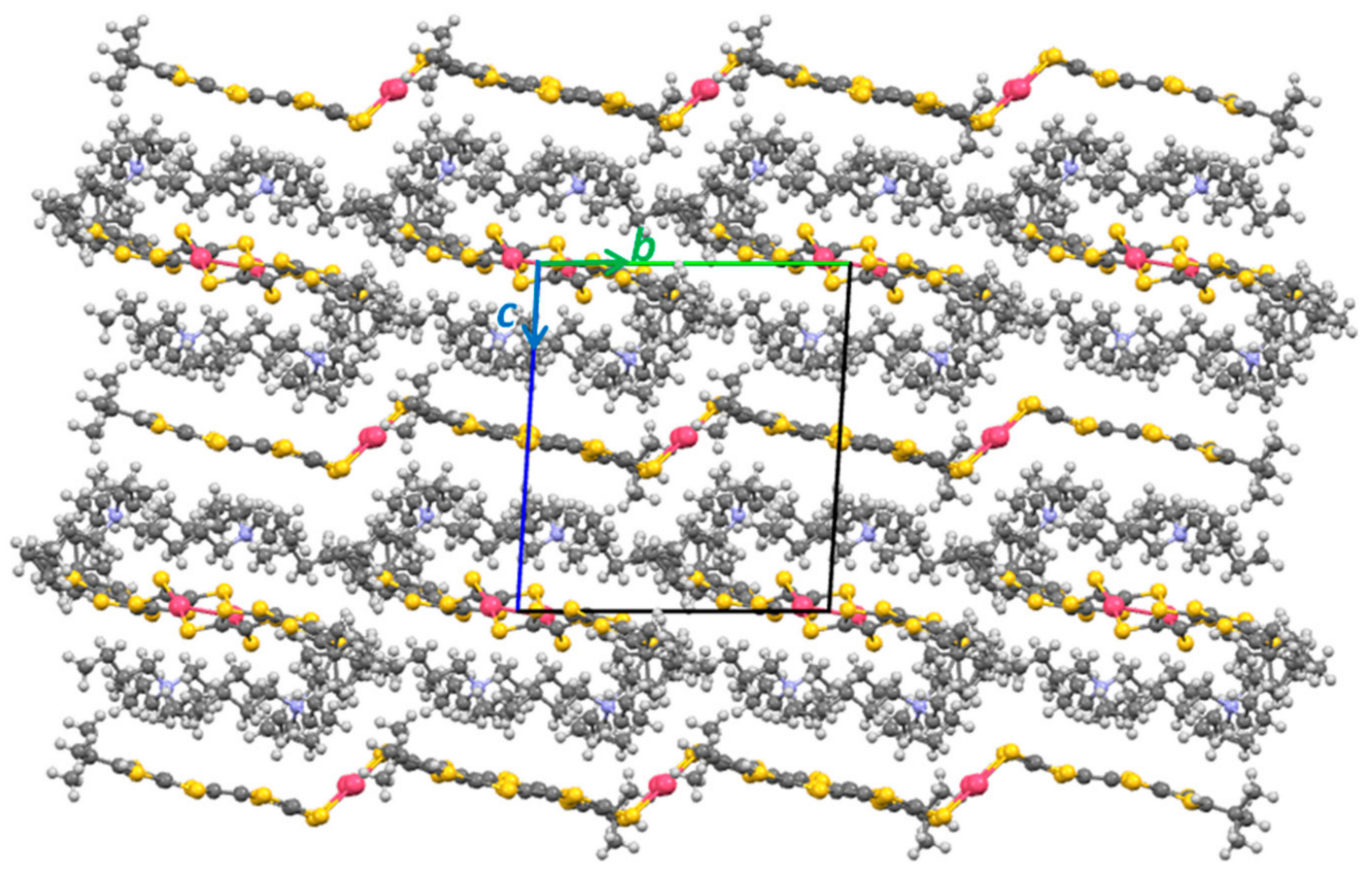
(*n*-Bu_4_N)_2_[Au_2_(α-tbtdt)_2_] (**5**) crystal structure viewed along the *a*-axis.

Figure 5 shows the crystal structure of compound **5** being composed of alternating monoanionic and cationic layers with a packing pattern similar to compound **3** and to related compounds (*n*-Bu_4_N)[Au(dtdt)_2_] and (*n*-Bu_4_N)[M(α-tdt)_2_] (M = Au, Ni) [17]. However, unlike related structures, compound **5** presents a gold-gold bond within the anionic species and, instead of being parallel, the relative position of the long axis of molecules, in consecutive anionic layers, are rotated ≈67°. To the best of our knowledge this is the first dinuclear gold (I) with this type of extended ligands. In these two molecules, Au1 and Au2, there are two gold atoms homo-bridged with a separation distance respectively of 3.0929(6) and 3.0137(4) Å, well within the range of the aurophilic bonding (2.50–3.50 Å) [22]. The sulfur coordination bond to the gold atoms is almost linear with S-Au-S angles of 168.83° for Au1 and 166.06° for Au2.

**Figure 6:**
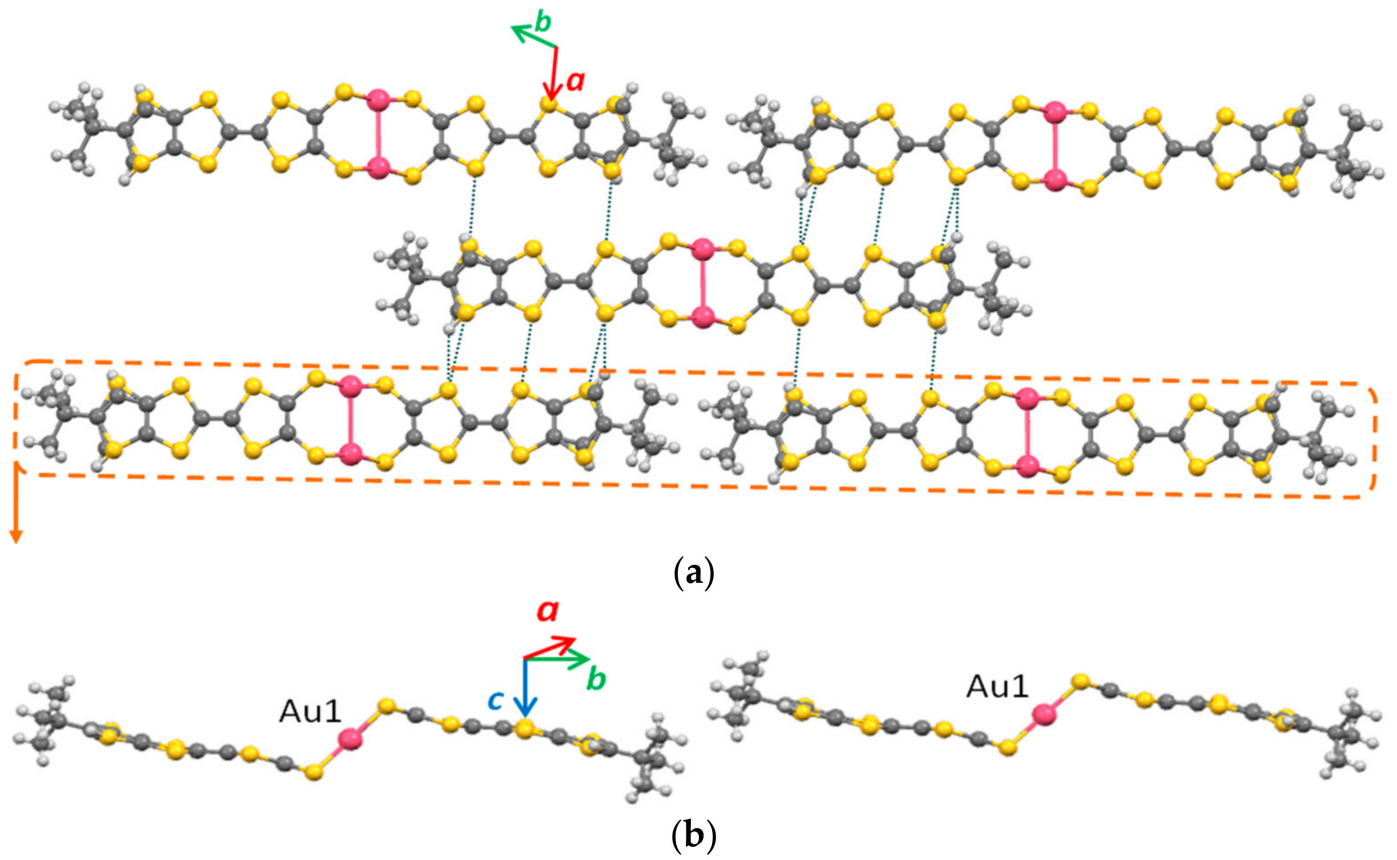
Anions Au1 in the crystal structure of (*n*-Bu_4_N)_2_[Au_2_(α-tbtdt)_2_] (**5**): (**a**) View perpendicular to one anionic layer with side-by-side contacts; (**b**) view perpendicular to anionic chains.

**Figure 7:**
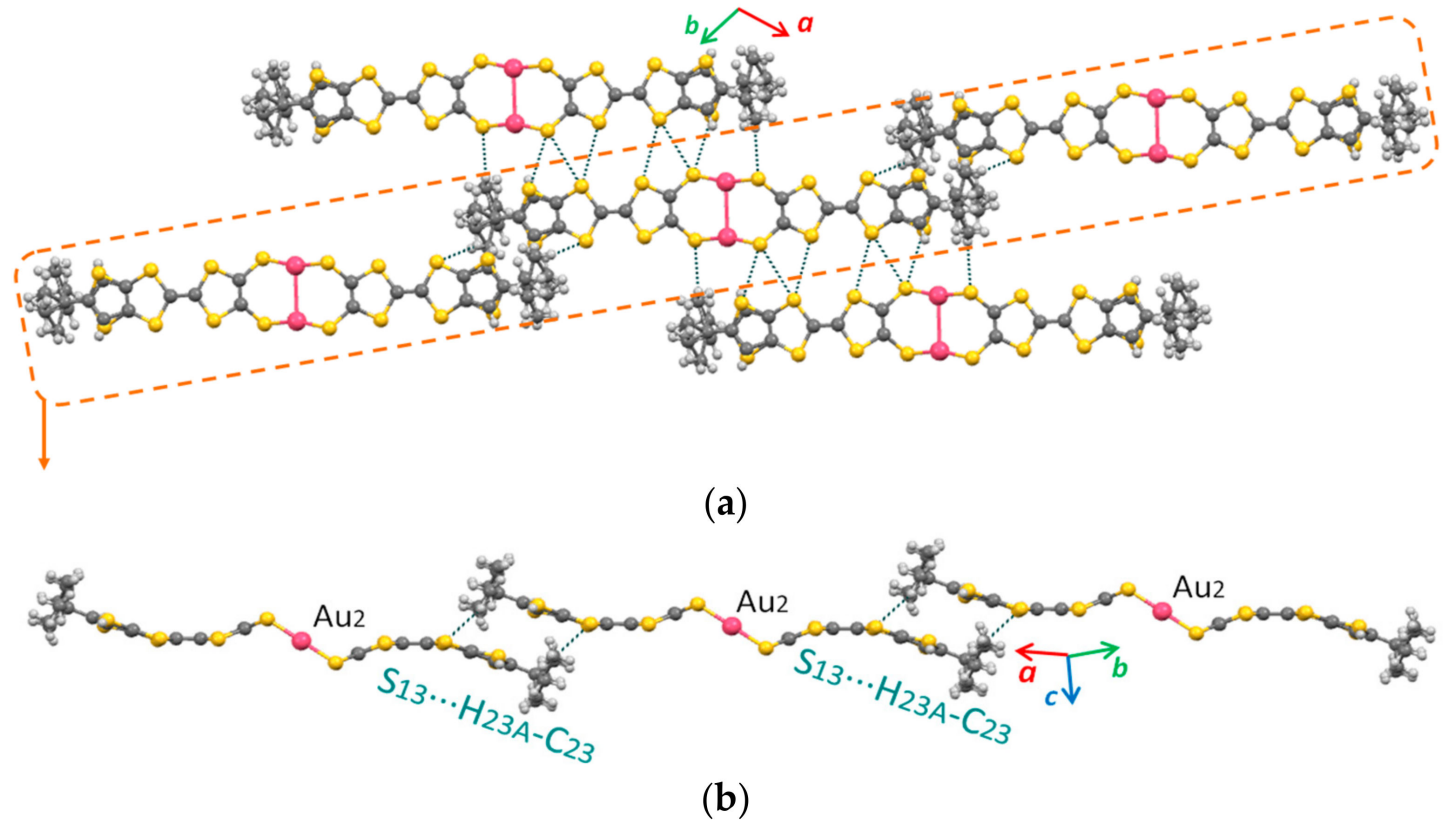
Anions Au2 in the crystal structure of (*n*-Bu_4_N)_2_[Au_2_(α-tbtdt)_2_] (**5**): (**a**) View perpendicular to one anionic layer with side-by-side contacts; (**b**) view perpendicular to anionic chains.

In compound **5** there are two alternating type of anionic layers, one composed of chains of molecules Au1 and another of chains of molecules Au2 (Figure 6 and Figure 7, respectively). Within these monoanionic layers the intermolecular contacts are different. The layers composed of Au2 anions have a larger number of side-by-side short S···S contacts and S···H-C hydrogen bonds (Figure 7) than the layers composed of Au1 (Figure 6). Due to the bulky *tert*-butyl groups that prevent a closer contact between molecules, in **5** the monoanionic [Au_2_(α-tbtdt)_2_] units in each layer are clearly more isolated than the ones in **3** ([Au(α-mtdt)_2_]). Nevertheless, in the layers composed by Au2 molecules an effective domino-like chain can be observed and the overlap along the molecules longest axis becomes clear by the hydrogen bond S13···H23–C23. Between cationic and anionic layers there are several S···H–C hydrogen bonds (Table S5).

### 2.4. Electric Transport Properties

In spite of the slightly higher solubility of the neutral compounds **6** and **7** when compared to related neutral complexes [M(dtdt)_2_] and [M(α-tdt)_2_] [17], no single crystals suitable for electrical transport properties could be isolated and therefore the electric transport properties of these compounds were measured in compressed polycrystalline powder pellets. The electrical resistivity measurements in these samples are dominated by interparticle resistance possibly further enhanced by anisotropy effects.

**Figure 8:**
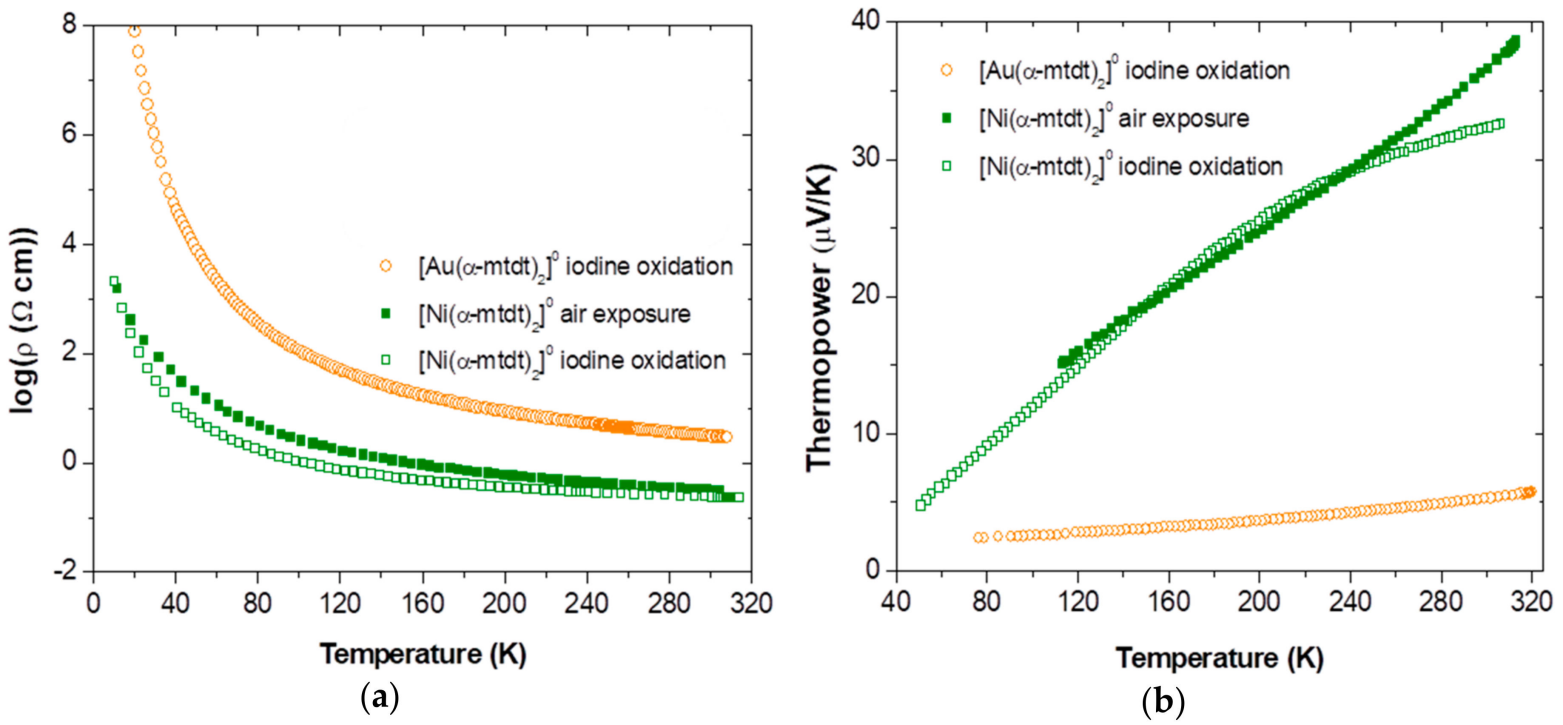
Electrical resistivity *π* (**a**) and absolute thermoelectric power *S* (**b**) of the neutral complexes [M(α-mtdt)_2_]° (M = Au and Ni) as a function of absolute temperature *T*.

Figure 8 presents the electrical resistivity (*ρ*) and absolute thermoelectric power (*S*) measured in polycrystalline samples of [M(α-mtdt)_2_] complexes with M = Au and Ni (**6** and **7,** respectively), obtained through air exposure (M = Ni) and by iodine solution (M = Au and Ni) as a function of temperature. All compounds display a typical semiconducting behavior with the resistivity increasing upon cooling (Figure 8a). This behavior was expected due to the interparticle resistance effects that dominate measurements in compressed powder samples. Nevertheless, the conductivity in the Ni complex (**7**) is higher than in the Au complex (**6**), and moreover it also exhibits lower activation energy (Table 1). All samples present conductivity values that are quite large for powder pellets and for compounds based on a neutral component. Measurements in different molecular conductors showed that electrical conductivity values obtained in powder pellets are usually 10^−2^ to 10^−3^ smaller than that of single crystal along its more conductive direction [1]. Therefore the low activation energies observed (54 meV and ≈26 meV for **6** and **7**, respectively) reflect most probably the interparticle resistance effects and not the intrinsic properties of the crystals.

The thermopower measurements (Figure 8b), which are not so sensitive to interparticle resistance effects and probe the intrinsic properties of the grains and their better conducting parts, show relatively small values (≈5 µV/K for [Au(α-mtdt)_2_] (**6**) and ≈32 µV/K for [Ni(α-mtdt)_2_] (**7**)) which decrease towards zero upon cooling providing a clear indication that the samples are metallic with the Fermi level lying in a continuum of states. The resistivity and thermoelectric power of the neutral [Ni(α-mtdt)_2_] complex (**7**) does not depend on the oxidation process since similar results were obtained in samples prepared either by air exposure or by iodine oxidation, in contrast to other related compounds.

Table 1 shows that, when compared to related compounds, [M(α-mtdt)_2_] complexes **6** and **7** present lower room temperature conductivity and slightly higher activation energy, which is probably due to the bulky methyl group that prevents stronger intermolecular interactions. The room temperature conductivity value for the tetrabutylammonium salt (*n*-Bu_4_N)[Au(α-mtdt)_2_] (1.5 × 10^−5^ S/cm) was found to be relatively large for a monoanionic (*n*-Bu_4_N) salt [10].

### 2.5. Magnetic Properties

**Figure 9:**
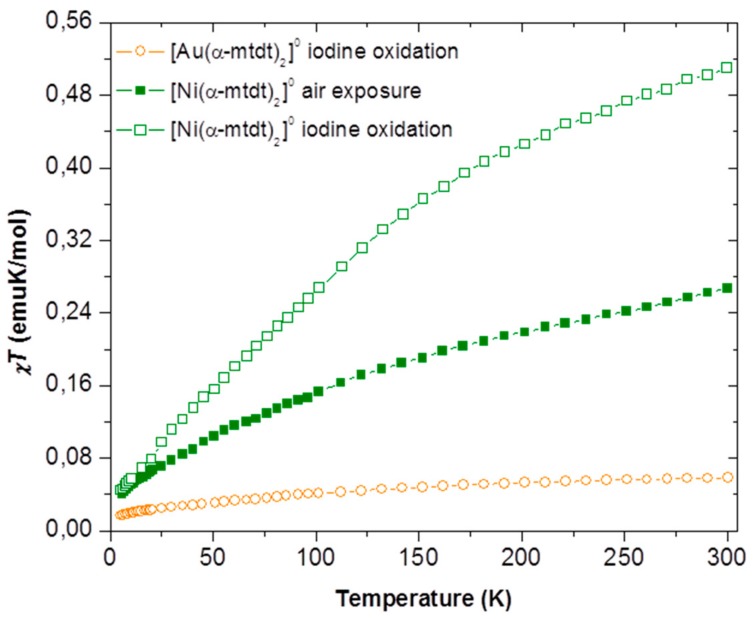
Temperature dependence of the χ*T* product for [M(α-mtdt)_2_] (M = Au (**6**) and Ni (**7**)) as a function of the absolute temperature *T*.

Figure 9 represents the static magnetic susceptibility of [M(α-mtdt)_2_] neutral complexes **6** and **7** in the temperature range 5–300 K. In all compounds a clear paramagnetic behavior was observed. Despite having an odd number of electrons [Au(α-mtdt)_2_] complex **6** presents very small values of magnetic susceptibility, being almost temperature independent over a wide temperature range. This type of behavior is ascribed to a Pauli-type contribution to the paramagnetic susceptibility typical of metallic systems [1]. For the neutral [Ni(α-mtdt)_2_] complex (**7**), with an even number of electrons, a diamagnetic behavior should have been expected. However, and as previously observed in related compounds [Ni(dtdt)_2_] and [Ni(α-tdt)_2_] [1,10,17], the magnetic measurements of complex **7**, obtained either by oxidation with iodine or through air exposure, clearly denote a paramagnetic behavior. Nevertheless, the magnetic susceptibility curves of these two nickel samples show very different progresses as the temperature increases reaching distinct χ*T* values at room temperature: The sample obtained by iodine oxidation reaches a value of 0.50 emuK/mol, significantly higher than the calculated for S = ½ (0.37 emuK/mol), while the sample obtained by air exposure has χ*T* = 0.26 emuK/mol. The reason for this different behavior between the samples is not yet clearly understood, although the large magnetic susceptibilities obtained for a supposedly neutral compound suggest that, in addition to the delocalized conduction electrons, some unpaired localized electrons can also be present and that these complexes can even exist in a high spin state [10].

## 3. Materials and Methods

### 3.1. General Information

All manipulations were carried out under strict anaerobic conditions under dry nitrogen, unless otherwise stated. All solvents were purified following the standard procedures [24]. Compounds **1** and **2**, pre-α-mtdt and pre-α-tbtdt, were synthesized as previously described [20]. Other chemicals were commercially obtained and used without any further purification. Elemental analyses of the compounds were performed using an EA 110 CE Instruments automatic analyzer (Wigan, UK). Melting points were studied on a Stuart Scientific SMP2 (Staffordshire, UK). IR spectra were obtained on a Bruker FTIR Tensor 27 spectrophotometer (Massachusetts, USA).

### 3.2. Synthesis

*Tetrabutylammonium salt of Gold (III) bis-2-(5-Methylthieno[2,3-d][1,3]dithiol-2-ylidene)-1,3-dithiole-4,5-dithiolate (3), (n-Bu_4_N)[Au(α-mtdt)_2_]*. Compound **1** (100 mg, 0.225 mmol) was added to a solution of sodium methoxide (0.1080 g, 2 mmol) in methanol (10 mL). After stirring for 1 h the orange solution was filtered and it is added dropwise potassium tetrachloroaurate (43 mg, 0.114 mmol) dissolved in methanol (2 mL). An hour later the inorganic impurities are filtered and to the filtered solution it was added dropwise tetrabutylammonium bromide (36.7 mg, 0.114 mmol) dissolved in methanol (1 mL). Immediately a golden precipitate forms and after a while the reaction mixture is filtered and the solid washed with methanol. Yield 51%. m.p.:105–109 °C. Elemental anal. calcd. for **3** (%): C_34_H_44_AuNS_14_, calcd.: C 36.71, H 3.99, N 1.26, S 40.34; found: C 37.02, H 3.90, N 1.40, S 41.02. FTIR(KBr), cm^−1^: 670 (m, HC=CH ligand), 1464 (m, thiophenic ring), 1473 (m, C–H aliph.), 2849 (m, C–H aliph.), 2917 (m, C–H aliph.).

*Gold (IV) bis-2-(5-Methylthieno[2,3-d][1,3]dithiol-2-ylidene)-1,3-dithiole-4,5-dithiolate (6), [Au(α-mtdt)_2_].* In a test tube compound **3** (20 mg, 1.80 × 10^−2^ mmol) was dissolved in dimethylformamide (2 mL). Similar to a diffusion cell it was slowly added a layer of acetonitrile (1 mL) followed by a solution of iodine (4.5 mg, 1.80 × 10^−2^ mmol) in acetonitrile (2 mL). The diffusion occurs slowly and a week later a dark thin powder is isolated by centrifugation, washed with acetone and dried. Yield 75%. m.p. > 370 °C. Elemental anal. calcd. for **6** (%): C_18_H_8_AuNS_14_, calcd.: C 24.85, H 0.93, S 51.59; found: C 22.76, H 0.85, S 48.40. FTIR(KBr), cm^−1^: 599 (w, C–S), 651 (m, HC=CH ligand), 1471 (m, thiophenic ring), 1634 (m, C=C).

*Bistetrabutylammonium salt of Nickel bis-2-(5-Methylthieno[2,3-d][1,3]dithiol-2-ylidene)-1,3-dithiole-4,5-dithiolate* (**4**)*, (n-Bu_4_N)_x_[Ni(α-mtdt)_2_] (x = 1,2)*. This compound was prepared following the same procedure as for **3**, using NiCl_2_·6H_2_O (27.1 mg, 0.114 mmol) instead of KAuCl_4_ and by doubling the tetrabutylammonium bromide quantity (73.5 mg, 0.228 mmol). The product obtained presented small needle shaped crystals which were agglomerated in a type of lace shape. This dark reddish shape could also be obtained through recrystallization in dichloromethane. Yield 50%. m.p.:150–154 °C. Elemental anal. calcd. for **4** (%): C_34_H_44_NiNS_14_, calcd.: C 41.92, H 4.55, N 1.44, S 46.07; C_50_H_80_NiN_2_S_14_, calcd.: C 49.36, H 6.63, N 2.30, S 36.89; found: C 43.84, H 5.20, N 1.89, S 41.81. FTIR(KBr), cm^−1^: 664 (m, HC=CH ligand), 1301 (s, Ni–S), 1459 (m, thiophenic ring), 1475 (m, C–H aliph.), 2850 (m, C–H aliph.), 2917 (m, C–H aliph.).

*Nickel (IV) bis-2-(5-Methylthieno[2,3-d][1,3]dithiol-2-ylidene)-1,3-dithiole-4,5-dithiolate* (**7**)*, [Ni(α-mtdt)_2_].* Compound **7** could be obtained through compound **4** air exposure or by the addition of an iodine solution. By air exposure, compound **4** (21.5 mg, 2.2 × 10^−2^ mmol) was dissolved in a test tube with 6 mL of dimethylformamide. The tube is then sealed and a small perforation was made upon its extraction from the glove box where it is left under aerobic conditions. Approximately two weeks later the dark crystalline thin powder is isolated by centrifugation, washed with acetone and dried. By the addition of an iodine solution it follows the same procedure described for **6**, in which compound **4** (14.8 mg, 1.52 × 10^−2^ mmol) was dissolved in dimethylformamide (5 mL) and after the layer of acetonitrile (1 mL) the solution of iodine (3.9 mg, 1.52 × 10^−2^ mmol) was added dropwise. A week later the same dark crystalline thin powder was obtained. Yield 14% (air exposure) and 35% (iodine solution). m.p. > 370 °C. Elemental anal. calcd. for **7** (%): C_18_H_8_NiS_14_, calcd.: C 29.54, H 1.10, S 61.3; found: (air exposure) C 30.07, H 1.54, S 59.08; (iodine solution) C 29.33, H 1.11, S 60.97. FTIR(KBr), cm^−1^: 669 (m, HC=CH ligand), 1397 (s, Ni–S), 1473 (m, thiophenic ring), 1637 (m, C=C).

*Tetrabutylammonium salt of dinuclear Gold (I) bis-2-(5-Methylthieno[2,3-d][1,3]dithiol-2-ylidene)-1,3-dithiole-4,5-dithiolate* (**5**)*, (n-Bu_4_N)_2_[Au_2_(α-tbtdt)_2_]*. This compound was prepared following the same method described for compound **3**, using as initial reactive compound **2** instead of **1**. Yield 15%. m.p.: 219 °C. Elemental anal. calcd. for **5** (%): C_56_H_92_Au_2_N_2_S_14_, calcd.: C 41.11, H 5.67, N 1.71, S 27.43; found: C 40.84, H 5.23, N 1.51, S 26.23. FTIR(KBr), cm^−1^: 668 (m, HC=CH ligand), 1457 (m, thiophenic ring), 1472 (m, C–H aliph.), 2870 (m, C–H aliph.), 2959 (m, C–H aliph.).

### 3.3. Cyclic Voltammetry

Cyclic voltammetry data were obtained using a sealed BAS C3 Cell Stand. The voltammograms were obtained at room temperature with variable scan rates in the range of 20–500 mV/s, platinum wire working and counter electrodes and a Ag/AgNO_3_ (0.01 M AgNO_3_ and 0.1 M *n*-Bu_4_NPF_6_ in acetonitrile) reference electrode, which the Ag^+^ ion electrode was separated from the bulk solution by a VycorTM frit. The measurements were performed on fresh solutions with a concentration of 1 × 10^−3^ M, in dichloromethane, that contained *n*-Bu_4_NPF_6_ (1 × 10^−1^ M) as supporting electrolyte. Measured in the same conditions the ferrocene/ferrocenium (Fc/Fc^+^) couple has E_1/2_ = 266 mV vs. Ag/AgNO_3_.

### 3.4. X-ray Crystallography

X-ray diffraction studies were performed with a Bruker APEX-II CCD detector diffractometer using graphite monochromated MoK_α_ radiation (λ = 0.71073 Å), in the φ and ω scans mode. A semi empirical absorption correction was carried out using SADABS [25]. Data collection, cell refinement and data reduction were done with the SMART and SAINT programs [26]. The structures were solved by direct methods using SIR97 [27] and refined by fullmatrix least-squares methods using the program SHELXL97 [28] using the winGX software package [29]. Non-hydrogen atoms were refined with anisotropic thermal parameters whereas H-atoms were placed in idealized positions and allowed to refine riding on the parent C atom. Molecular graphics were prepared using ORTEP 3 [30].

**Crystallographic data for compound 3**: C_34_H_44_AuNS_14_, M = 1112.51 g·mol^−1^, triclinic, space group *P*-1, *a* = 8.7883(4) Å, *b* = 9.8828(4) Å, *c* = 25.2450(10) Å, α = 85.697(3)°, β = 82.933(2)°, γ = 78.627(2)°, V = 2130.31(15) Å^3^, Z = 2, π_calc_ = 1.734 g·cm^−3^, µ(Mo *K*α) = 4.167 mm^−1^, 15774 reflections measured, 7373 unique [R_int_ = 0.0549], θ_max_ = 25.03°, R1 = 0.0443 using 5177 Refl. > 2σ(I), ωR2 = 0.0667, T = 150(2) K. CCDC 1871868.

**Crystallographic data for compound 5**: C_56_H_92_Au_2_N_2_S_14_, M = 1636.08 g·mol^−1^, triclinic, space group *P*-1, *a* = 13.2047(3) Å, *b* = 16.0158(4) Å, *c* = 16.9624(4) Å, α = 90.921(2)°, β = 97.1910(10)°, γ = 109.3910(10)°, V = 3350.79(14) Å^3^, Z = 2, π_calc_ = 1.622 g.cm^−3^, µ(Mo *K*α) = 4.846 mm^−1^, 32406 reflections measured, 12652 unique [R_int_ = 0.0464], θ_max_ = 25.681°, R1 = 0.0558 using 8598 Refl. > 2σ(I), ωR2 = 0.1302, T = 150(2) K. CCDC 1817866.

### 3.5. Electric Transport Properties

Electrical conductivity and thermopower were measured in compressed pellets of polycrystalline samples in the temperature range of 50–320 K, using a measurement cell attached to the cold stage of a closed cycle helium refrigerator. In the first step, the thermopower was measured by using a slow AC (ca. 10^−2^ Hz) technique [31], by attaching two Ø = 25 μm diameter 99.99% pure Au wires (Goodfellow), thermally anchored to two quartz blocks, with Pt paint (Demetron 308A) to the extremities of an elongated sample as in a previously described apparatus [32], controlled by a computer [33]. The oscillating thermal gradient was kept below 1 K and was measured with a differential Au-0.05 at. % Fe vs. chromel thermocouple of the same type. The absolute thermoelectric power of the samples was obtained after correction for the absolute thermopower of the Au leads, by using the data of Huebner [34]. In a second step two additional contacts were placed to achieve a four-in-line contact configuration to perform electrical resistivity measurements. In the case of more conducting samples a low-frequency four-probe AC method (77 Hz) was used, with a SRS Model SR83 Lock-in Amplifier and applying a 5 µA current; for more resistive samples a four-probe DC method was used instead, using a Keithley 224 current source to apply both direct and reverse DC currents, well below 0.1 μA, through the sample and Keithley 619 electrometer to measure the corresponding DC voltage.

### 3.6. Magnetic Susceptibility

The magnetic susceptibility was measured with a S700X SQUID magnetometer (Cryogenic Ltd., London, UK) in the temperature range 5–300 K under a static magnetic field of 1 T. The paramagnetic susceptibility of the different samples was assumed after correction for the sample holder and core diamagnetism estimated from tabulated Pascal constants, as −329.2 × 10^−6^ emu/mol and −309.2 × 10^−6^ emu/mol for compounds **6** and **7**, respectively.

## 4. Conclusions

In conclusion, new monoanionic and neutral nickel and gold complexes based on extended thiophenic-TTF fused bisdithiolene ligands substituted with alkyl groups were prepared. When compared to their unsubstituted counterparts and as expected, the incorporation of methyl and *tert*-butyl groups in the thiophenic ring led to an increase of solubility in common organic solvents. In the methyl substituted nickel and gold neutral complexes, [M(α-mtdt)_2_] (**6**–**7**), the conductivity was not substantially affected by the introduction of the alkyl group, where a highly conducting behavior, in polycrystalline samples, could be found ([Au(α-mtdt)_2_] = 0.32 S/cm and [Ni(α-mtdt)_2_] ≈ 4 S/cm) and still are compatible with metallic properties. The metallic properties are further supported by the small thermoelectric power, decreasing upon cooling almost linearly with temperature. The synthetic procedure with the *tert*-butyl substituent leads to a Au (I) dinuclear complex, (*n*-Bu_4_N)_2_[Au_2_(α-tbtdt)_2_] (**5**), instead of the expected mononuclear Au(III) complex. In this monoanionic compound the large bulky *tert*-butyl group prevents short S···S interactions and a more efficient intermolecular overlap.

The preparation of methyl substituted gold and nickel thiophenetetrathiafulavalenedithiolate complexes [M(α-mtdt)_2_] enlarged the family of single component molecular conductors. Due to their slightly higher solubility, which enables them to be processed using solution techniques, they are good candidates for conducting and magnetic materials, as well as building blocks in electronic devices.

## Figures and Tables

**Table 1:** Room-temperature electrical conductivity (σ _RT_) and activation energy (E_a_) for [M(α-mtdt)_2_] (M = Au and Ni) and related compounds.

Complexes	Oxidation Process	σ_RT_	E_a_
		(S/cm)	(meV)
(*n*-Bu_4_N)[Au(α-mtdt)_2_] [23]	−	1.5 × 10^−5^	143
[Au(α-mtdt)_2_] [23]	Iodine	0.32	54
[Ni(α-mtdt)_2_] [23]	Iodine	4.2	20
	Air	3.2	33
[Au(α-tdt)_2_] [17]	Iodine	5	<20
[Au(dtdt)_2_] [17]	Iodine	8	<20
[Ni(α-tdt)_2_] [17]	Iodine	24	<20
	Air	2	<20
[Ni(dtdt)_2_] [17]	Iodine	200	<20
	Air	2.5	<20

## Supplementary Materials

CCDC 1817866–1817868 contains the supplementary crystallographic data for this paper. These data can be obtained free of charge via http://www.ccdc.cam.ac.uk/conts/retrieving.html. The following are available online, Figure S1: Cyclic voltammetry of compounds **3** and **4**, Figure S2: Cyclic voltammetry of compound **5**, Figure S3: X-ray powder pattern of compound **4** and X-ray powder pattern simulation of compound **3**, X-Ray structural analysis of ligand precursor **1**, Figure S4: Crystal structure of compound **1**, Table S1: Bond lengths of compound **3**, Table S3 and Table S4: Bond lengths of compound **5**, Table S2 and Table S5: Short contacts of compounds **3** and **5**, respectively.
