# Supplementary material for: Gold and Nickel Extended Thiophenic-TTF Bisdithiolene Complexes
Source: Molecules. 2018 Feb 14;23(2):424. doi: 10.3390/molecules23020424 (PMC6017561; doi:10.3390/molecules23020424)
Supplement: Supplementary file 1 [file molecules-23-00424-s001.pdf]

Gold and Nickel Extended Thiophenic—TTF Bisdithiolene Complexes

Rafaela A. L. Silva, Bruno J. C. Vieira, Marta M. Andrade, Isabel C. Santos, Sandra Rabaça, Elsa B. Lopes, Joana T. Coutinho, Laura C. J. Pereira, Manuel Almeida and Dulce Belo *

C[2]TN, Centro de Ciências e Tecnologias Nucleares, Instituto Superior Técnico, Universidade de Lisboa, E.N. 10 ao km 139.7, 2695-066 Bobadela LRS, Portugal; rafaela@ctn.tecnico.ulisboa.pt (R.A.L.S.); brunovieira@ctn.tecnico.ulisboa.pt (B.J.C.V.); sandrar@ctn.tecnico.ulisboa.pt (S.R.); eblopes@ctn.tecnico.ulisboa.pt (E.B.L.); coutinho.joana@ctn.tecnico.ulisboa.pt (J.T.C.); lpereira@ctn.tecnico.ulisboa.pt (L.C.J.P.); malmeida@ctn.tecnico.ulisboa.pt (M.A.)

* Correspondence: dbelo@ctn.tecnico.ulisboa.pt; Tel.: +351-21-955-6203

Supplementary Materials

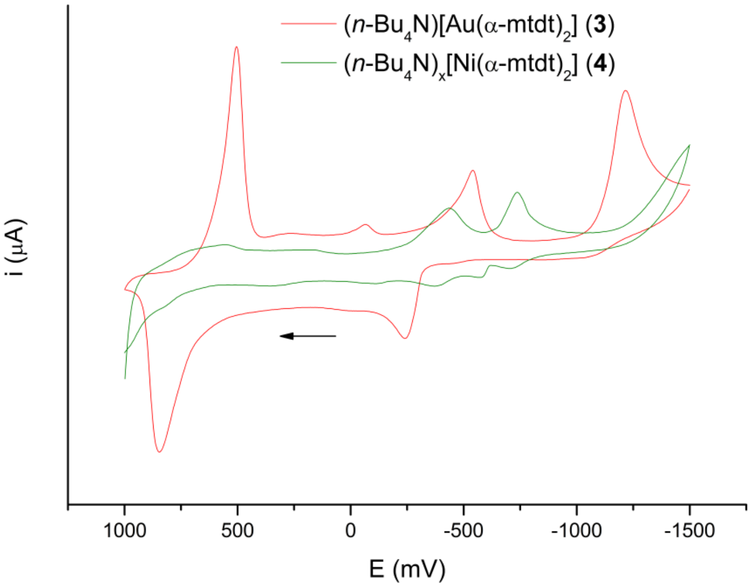

Figure S1. Cyclic voltammetry of (*n*-Bu_4N)[Au(α-mtdt)$_2$] (**3**) and (*n*-Bu_4N)$_x$[Ni(α-mtdt)$_2$] (**4**) vs Ag/$AgNO_3$ (measured in the same conditions the Fc/Fc^+ couple has a $E_{1/2}$ = 266 mV).

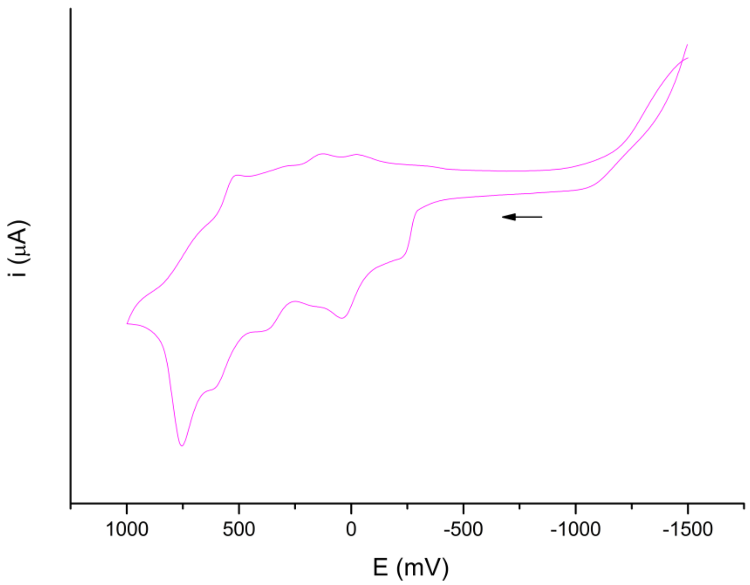

Figure S2. Cyclic voltammetry of (*n*-$Bu_4N)_2[Au_2(\alpha$-tbtdt$)_2]$ (**5**) vs. Ag/$AgNO_3$ (measured in the same conditions the Fc/Fc^+ couple has a $E_{1/2}$ = 266 mV).

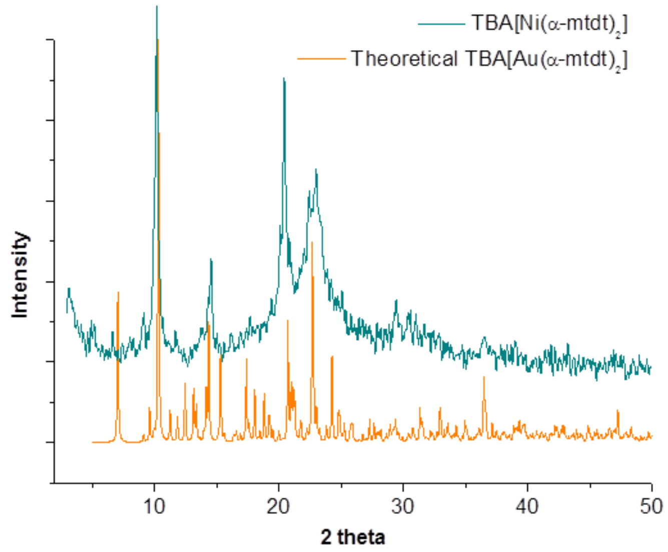

Figure S3. X-ray powder pattern of (*n*-$Bu_4N)_x[Ni(\alpha$-mtdt$)_2]$ (**4**, green line) and X-ray powder pattern simulation from (*n*-$Bu_4N)[Au(\alpha$-mtdt$)_2]$ (**3**, orange line) crystal structure.

X-Ray Structural Analysis of Ligand Precursor 1

Here we will report the crystal structure of ligand precursor **1**, which its preparation and characterization was previously reported however no X-ray structure could be determined [1].

Ligand precursor **1** crystallizes in the monoclinic system, space group $P2_1$. The asymmetric unit cell contains two independent neutral α-mtdt molecules, both at general positions. These two α-mtdt molecules are essentially planar, with exception of the cyanoethyl groups, and present disorder in the thiophenic ring with sulphur atoms over two possible positions with occupation factors of 44–56 % (S1/C3-S1A/C3A) for one molecule while the other has 53–47 % (S8/C18-S8A/C18A).

Ligand precursor **1** crystallizes as fibers with very small dimensions severely limiting the X-ray diffraction analysis. Nevertheless, and although the quality of the data collected did not allow a good structural refinement it is consistent with other extended thiophenic-TTF fused dithiolene ligands like the unsubstituted non-aromatic dtdt [2] and aromatic α-tdt [2] and *tert*-butyl-substituted pre-α-tbtdt [1]. Compound **1** crystallizes in the monoclinic system, space group $P2_1$, and it is composed of layers of side-by-side A-B-A-B chains of molecules, running parallel to *c* (Figure S4a). Within the chain the molecules are connected by several S···S short contacts and arranged in a head-to-tail fashion where the cyanoethyl group points outside the chain and the mean plane of adjacent molecules is rotated by about 50°. Along *a*, the chains are regularly packed and connected by C-H···S and C-H···N hydrogen bonds (Figure S4b). Between layers, molecules are connected by several C-H···N hydrogen bonds.

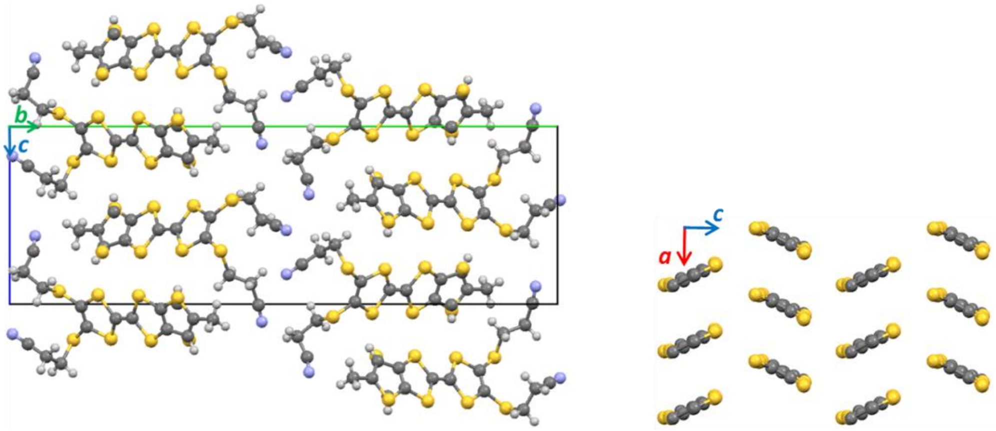

(**a**) (**b**)

Figure S4. Crystal structure of compound **1**: (**a**) View along the *a* axis; (**b**) partial view along *b* of a layer showing the tilt between the molecules relatively to the chain axis *a* (the cyanoethyl group and hydrogen atoms were omitted for clarity).

Crystallographic data for compound 1: $C_{30}H_{24}N_4S_{14}$, M=889.37 g.mol^{-1}, monoclinic, space group P2$_1$, *a* = 5.1144(3)Å, *b* = 33.259(2)Å, *c* = 10.7953(7)Å, β = 90.104(4)°, V = 1836.3(2)Å^3, Z = 2, ρ_{calc} = 1.608 g.cm^{-3}, μ(Mo *K*α) = 0.859 mm^{-1}, 12015 reflections measured, 6023 unique [R_{int}=0.0511], θ_{max} = 25.026°, R1 = 0.0983 using 4256 Refl.>2σ(I), *ω*R2 = 0.2661, T = 150(2) K. CCDC 1817867.

---

1. Silva, R. A. L.; Vieira, B. J. C.; Andrade, M. A.; Santos, I. C.; Rabaça, S.; Belo, D.; Almeida, M. TTFs nonsymmetrically fused with alkylthiophenic moieties. *Beilstein J. Org. Chem.* **2015**, *11*, 628-637, DOI: 10.3762/bjoc.11.71.
2. Belo, D.; Figueira, M. J.; Nunes, J. P. M.; Santos, I. C.; Almeida, M.; Crivillers, N.; Rovira, C. Synthesis and characterization of the novel extended TTF-type donors with thiophenic units. *Inorg. Chim. Acta* **2007**, *360*, 3909-3914, DOI: 10.1016/j.ica.2007.03.041.

Table S1. Bond lengths in the crystal structure of (n-Bu$_4$N)[Au(α-mtdt)$_2$] (**3**).

**(n-Bu$_4$N)$^+$**	**d (Å)**	**[Au(α-mtdt)$_2$]$^-$**	**d (Å)**
C19-C20	1.530(10)	Au1-S8	2.3069(18)
C19-H19A	0.99	Au1-S2	2.3071(17)
C19-H19B	0.99	Au1-S9	2.326(2)
C20-C21	1.492(10)	Au1-S1	2.3262(19)
C20-H20A	0.99	S1-C1	1.764(6)
C20-H20B	0.99	S2-C2	1.753(7)
C21-C22	1.508(11)	S3-C1	1.764(7)
C21-H21A	0.99	S3-C3	1.767(7)
C21-H21B	0.99	S4-C2	1.756(6)
C22-H22A	0.98	S4-C3	1.769(7)
C22-H22B	0.98	S5-C6	1.743(7)
C22-H22C	0.98	S5-C4	1.770(7)
C23-C24	1.525(10)	S6-C5	1.742(8)
C23-H23A	0.99	S6-C4	1.757(7)
C23-H23B	0.99	S8-C10	1.748(8)
C24-C25	1.502(10)	S9-C11	1.752(7)
C24-H24A	0.99	S10-C12	1.766(8)
C24-H24B	0.99	S10-C10	1.781(6)
C25-C26	1.527(10)	S11-C11	1.759(7)
C25-H25A	0.99	S11-C12	1.772(7)
C25-H25B	0.99	S12-C15	1.759(8)
C26-H26A	0.98	S12-C13	1.780(7)
C26-H26B	0.98	S13-C14	1.748(7)
C26-H26C	0.98	S13-C13	1.754(8)
C27-C28	1.537(11)	N1-C23	1.511(9)
C27-H27A	0.99	N1-C27	1.511(10)
C27-H27B	0.99	N1-C19	1.518(9)
C28-C29	1.482(12)	N1-C31	1.533(10)
C28-H28A	0.99	C1-C2	1.326(9)
C28-H28B	0.99	C3-C4	1.337(9)
C29-C30	1.503(12)	C5-C6	1.360(10)
C29-H29A	0.99	C5-C7A	1.428(19)
C29-H29B	0.99	C5-S7	1.766(8)
C30-H30A	0.98	C6-C7	1.498(18)
C30-H30B	0.98	C6-S7A	1.722(9)
C30-H30C	0.98	C8-C7	1.321(18)
C31-C32A	1.492(16)	C8-C7A	1.37(2)
C31-C32	1.564(15)	C8-C9	1.507(9)
C31-H31A	0.99	C8-S7	1.703(9)
C31-H31B	0.99	C8-S7A	1.720(9)

Table S1. Bond lengths in the crystal structure of (*n*-Bu_4N)[Au(α-mtdt)$_2$] (**3**). (cont.)

(*n*-Bu_4N)$^+$	d (Å)
C34-C33	1.441(13)
C34-H34A	0.98
C34-H34B	0.98
C34-H34C	0.98
C33-C32	1.255(15)
C33-C32A	1.406(17)
C33-H33A	0.99
C33-H33B	0.99
C32-H32B	0.99
C32A-H32C	0.99
C32A-H32D	0.99

[Au(α-mtdt)$_2$]$^-$	d (Å)
C9-H9A	0.98
C9-H9B	0.98
C9-H9C	0.98
C10-C11	1.305(9)
C12-C13	1.333(9)
C14-C15	1.333(10)
C14-C16A	1.63(2)
C14-S14	1.695(10)
C15-S14A	1.643(10)
C15-C16	1.643(19)
C17-C16	1.48(2)
C17-C18	1.522(11)
C17-S14	1.556(11)
C17-C16A	1.557(19)
C17-S14A	1.567(12)
C18-H18A	0.98
C18-H18B	0.98
C18-H18C	0.98
C7-H7	0.95
C16-H16	0.95
C7A-H7A	0.95
C16A-H16A	0.95
C32-H32A	0.99

Table S2. Short S···S and hydrogen bonds in the crystal structure of (*n*-Bu_4N)[Au(α-mtdt)$_2$] (**3**).

	Symm. op.*	Length (Å)	Contact type
S1···S6	x,-1+y,z	3.603(2)	M-M C
S8···S4	x,-1+y,z	3.540(2)	M-M C
S8···S6	x,-1+y,z	3.652(3)	M-M C
S10···S2	x,-1+y,z	3.553(2)	M-M C
S12···S2	x,-1+y,z	3.691(3)	M-M C
S12···S9	x,-1+y,z	3.555(3)	M-M C
S11···S13	-x,-y,-z	3.547(3)	M-M C
S13···S13	-x,-y,-z	3.542(3)	M-M C
S3···S3	-x,1-y,1-z	3.619(2)	M-M C
S4···H9A-C9	-x,2-y,1-z	2.968 (150.72 °)	M-M W
S4···H19A-C19	1-x,1-y,1-z	3.021 (123.52 °)	M-TBA
S5···S7	-x,2-y,1-z	3.605(5)	M-M W
C5···H9C-C9	1-x,2-y,1-z	2.821 (83.09 °)	M-M L

Table S2. Short S···S and hydrogen bonds in the crystal structure of (n-Bu_4N)[Au(α-mtdt)$_2$] (**3**). (cont.)

	Symm. op.*	Length (Å)	Contact type
S13···H34B-C34	-1+x,y,-1+z	3.012 (162.78 °)	M-TBA
S10···H25B-C25	-x,-y,1-z	3.032 (127.34 °)	M-TBA
S12···H27A-C27	-x,-y,1-z	2.922 (152.17 °)	M-TBA
S4···H25A-C25	-x,1-y,1-z	3.034 (160.39 °)	M-TBA
S10···H31A-C31	1-x,-y,1-z	3.078 (107.21 °)	M-TBA
S2···H31A-C31	1-x,1-y,1-z	2.953 (160.10 °)	M-TBA

C - Between chains in the same layer; L - Between chains in different layers; M - Monoanion [Au(α-mtdt)$_2$]; TBA - Cation (n-Bu_4N); W - Along a chain.

Table S3. Bond lengths relative to tetrabutylammonium molecules A and B in the crystal structure of (n-Bu_4N)$_2$[Au_2(α-tbtdt)$_2$] (**5**).

A	d (Å)	B	d (Å)
N1-C33	1.509(10)	N2-C45	1.503(10)
N1-C29	1.511(10)	N2-C53	1.504(10)
N1-C25	1.526(11)	N2-C49	1.509(10)
N1-C37	1.526(10)	N2-C41	1.516(9)
C25-C26	1.523(12)	C41-C42	1.505(12)
C25-H25A	0.9900	C41-H41A	0.9900
C25-H25B	0.9900	C41-H41B	0.9900
C26-C27	1.515(13)	C42-C43	1.476(14)
C26-H26A	0.9900	C42-H42A	0.9900
C26-H26B	0.9900	C42-H42B	0.9900
C27-C28	1.453(15)	C43-C44	1.451(17)
C27-H27A	0.9900	C43-H43A	0.9900
C27-H27B	0.9900	C43-H43B	0.9900
C28-H28A	0.9800	C44-H44A	0.9800
C28-H28B	0.9800	C44-H44B	0.9800
C28-H28C	0.9800	C44-H44C	0.9800
C29-C30	1.498(12)	C45-C46	1.502(12)
C29-H29A	0.9900	C45-H45A	0.9900
C29-H29B	0.9900	C45-H45B	0.9900
C30-C31	1.472(13)	C46-C47	1.502(13)
C30-H30A	0.9900	C46-H46A	0.9900
C30-H30B	0.9900	C46-H46B	0.9900
C31-C32	1.490(14)	C47-C48	1.508(14)
C31-H31A	0.9900	C47-H47A	0.9900
C31-H31B	0.9900	C47-H47B	0.9900
C32-H32A	0.9800	C48-H48A	0.9800
C32-H32B	0.9800	C48-H48B	0.9800
C32-H32C	0.9800	C48-H48C	0.9800

Table S3. Bond lengths relative to tetrabutylammonium molecules A and B in the crystal structure of (n-$Bu_4N)_2[Au_2$(α-tbtdt$)_2$] (**5**). (cont.)

A	d (Å)
C33-C34	1.487(13)
C33-H33A	0.9900
C33-H33B	0.9900
C34-C36A	1.41(3)
C34-C35	1.61(3)
C34-H34A	0.9900
C34-H34B	0.9900
C37-C38	1.522(12)
C37-H37A	0.9900
C37-H37B	0.9900
C38-C39	1.538(12)
C38-H38A	0.9900
C38-H38B	0.9900
C39-C40	1.523(14)
C39-H39A	0.9900
C39-H39B	0.9900
C40-H40A	0.9800
C40-H40B	0.9800
C40-H40C	0.9800
C35-C36	1.488(17)
C35-H35A	0.9900
C35-H35B	0.9900
C36-H36A	0.9800
C36-H36B	0.9800
C36-H36C	0.9800
C35A-C36A	1.44(4)
C35A-H35C	0.9800
C35A-H35D	0.9800
C35A-H35E	0.9800
C36A-H36A	0.9900
C36A-H36B	0.9900

B	d (Å)
C49-C50	1.519(11)
C49-H49A	0.9900
C49-H49B	0.9900
C50-C51	1.504(12)
C50-H50A	0.9900
C50-H50B	0.9900
C51-C52	1.512(12)
C51-H51A	0.9900
C51-H51B	0.9900
C52-H52A	0.9800
C52-H52B	0.9800
C52-H52C	0.9800
C53-C54	1.530(12)
C53-H53A	0.9900
C53-H53B	0.9900
C54-C55	1.505(14)
C54-H54A	0.9900
C54-H54B	0.9900
C55-C56	1.517(16)
C55-H55A	0.9900
C55-H55B	0.9900
C56-H56A	0.9800
C56-H56B	0.9800
C56-H56C	0.9800

Table S4. Bond lengths of [Au_2(α-tbtdt)$_2$] molecules Au1 and Au2 in the crystal structure of (*n*-Bu_4N)$_2$[Au_2(α-tbtdt)$_2$] (**5**).

Au1	d (Å)	Au2	d (Å)
Au1-S2	2.272(2)	Au2-S8	2.279(2)
Au1-S1	2.284(2)	Au2-S9	2.279(2)
Au1-Au1	3.0929(7)	Au2-Au2	3.0137(6)
S1-C1	1.755(9)	S8-C13	1.769(8)
S2-C2	1.723(10)	S9-C14	1.747(8)
S2-Au1	2.272(2)	S9-Au2	2.279(2)
S3-C3	1.748(9)	S10-C13	1.744(8)
S3-C1	1.766(11)	S10-C15	1.750(7)
S4-C3	1.754(10)	S11-C15	1.738(9)
S4-C2	1.769(9)	S11-C14	1.778(7)
S5-C5	1.734(10)	S12-C16	1.766(9)
S5-C4	1.761(9)	S12-C17	1.778(8)
S6-C6	1.742(11)	S13-C18	1.753(9)
S6-C4	1.761(9)	S13-C16	1.764(8)
C1-C2	1.345(13)	C13-C14	1.335(11)
C3-C4	1.348(12)	C15-C16	1.342(11)
C5-C6	1.368(14)	C17-C18	1.316(11)
C5-C7	1.405(18)	C17-C19A	1.438(18)
C5-S7A	1.734(19)	C17-S14	1.677(11)
C6-S7	1.666(11)	C18-C19	1.53(2)
C6-C7A	1.95(3)	C18-S14A	1.749(9)
C8-C7	1.307(18)	C19-H19	0.9500
C8-C7A	1.52(3)	C19A-H19A	0.9500
C8-C9	1.505(13)	C20-C19	1.34(2)
C8-S7A	1.737(18)	C20-C19A	1.393(17)
C8-S7	1.756(11)	C20-C21	1.490(11)
C9-C11	1.515(14)	C20-S14A	1.684(10)
C9-C10	1.535(13)	C20-S14	1.688(10)
C9-C12	1.548(13)	C21-C23A	1.35(3)
C10-H10A	0.9800	C21-C24	1.388(19)
C10-H10B	0.9800	C21-C22	1.538(18)
C10-H10C	0.9800	C21-C24A	1.58(3)
C11-H11A	0.9800	C21-C23	1.635(17)
C11-H11B	0.9800	C21-C22A	1.79(2)
C11-H11C	0.9800	C22-H22A	0.9800

Table S4. Bond lengths of [Au_2(α-tbtdt)$_2$] molecules Au1 and Au2 in the crystal structure of (*n*-Bu_4N)$_2$[Au_2(α-tbtdt)$_2$] (**5**). (cont.)

Au1	d (Å)
C12-H12A	0.9800
C12-H12B	0.9800
C12-H12C	0.9800
C7-H7	0.9500
C7A-H7A	0.9500

Au2	d (Å)
C22-H22B	0.9800
C22-H22C	0.9800
C23-H23A	0.9800
C23-H23B	0.9800
C23-H23C	0.9800
C24-H24A	0.9800
C24-H24B	0.9800
C24-H24C	0.9800
C22A-H22D	0.9800
C22A-H22E	0.9800
C22A-H22F	0.9800
C23A-H23D	0.9800
C23A-H23E	0.9800
C23A-H23F	0.9800
C24A-H24D	0.9800
C24A-H24E	0.9800
C24A-H24F	0.9800

Table S5. Short S···S and hydrogen bonds in the crystal structure of (*n*-Bu_4N)$_2$[Au_2(α-tbtdt)$_2$] (**5**).

	Symm. op.*	Length (Å)	Contact type
S4···S7	-x,-y,1-z	3.669(6)	Au1-Au1 W
S4···H7A-C7A	-x,-y,1-z	2.89 (149.35 °)	Au1-Au1 W
S6···S6	-x,-y,1-z	3.542(4)	Au1-Au1 W
S3···S7A	1-x,-y,1-z	3.5858	Au1-Au1 W
S3···H29B-C29	x,y,z	2.895 (144.73 °)	Au1-TBA
S3···H31A-C31	x,y,z	2.989 (153.64 °)	Au1-TBA
S1···H27A-C27	x,y,z	2.977 (162.09 °)	Au1-TBA
S7A···H31A-C31	x,1+y,z	2.886 (130.44 °)	Au1-TBA
S3···H53A-C53	x,y,z	2.992 (133.18 °)	Au1-TBA
S5···H53B-C53	x,y,z	2.912 (139.13 °)	Au1-TBA
S7A···H56A-C56	x,1+y,z	2.815 (108.68 °)	Au1-TBA
S7A···H56B-C56	x,1+y,z	2.827 (107.86 °)	Au1-TBA
S8···S12	-x,-y,-z	3.563(3)	Au2-Au2 W
S8···S14	-x,-y,-z	3.631(6)	Au2-Au2 W
S10···S12	-x,-y,-z	3.650(3)	Au2-Au2 W
C22A-H22F···S9	-1+x,y,z	2.881 (107.27 °)	Au2-Au2 W
S13···H23D-C23	-x,1-y,-z	2.992 (149.64 °)	Au2-Au2 W

Table S5. Short S···S and hydrogen bonds in the crystal structure of (n-$Bu_4N)_2[Au_2$(α-tbtdt$)_2$] (**5**). (cont.)

	**Symm. op.***	**Length (Å)**	**Contact type**
Au2···H49B-C49	x,y,-1+z	2.873 (144.53 °)	Au2-TBA
S10···H49A-C49	x,y,-1+z	2.888 (158.65 °)	Au2-TBA
S11···H41A-C41	x,y,-1+z	2.834 (160.03 °)	Au2-TBA
Au2···H41B-C41	x,y,-1+z	2.831 (154.47 °)	Au2-TBA
S8···H54A-C54	x,y,-1+z	3.019 (146.95 °)	Au2-TBA
S14···H51B-C51	1+x,y,-1+z	2.941 (116.77 °)	Au2-TBA

TBA - Cation (n-Bu_4N); W - Along a chain
